# Supplementary material for: A low Smc flux avoids collisions and facilitates chromosome organization in Bacillus subtilis
Source: eLife. 2021 Aug 4;10:e65467. doi: 10.7554/eLife.65467 (PMC8357415; doi:10.7554/eLife.65467)
Supplement: Supplementary file 1. [file elife-65467-supp1.docx]

**Information about replicates for each relevant figure panel.**

As a biological replicate we define a sample that was processed independently beyond strain construction. For 3C-seq these are samples prepared from independent culturing events. For spotting it is either independent culturing event and/or two independently obtained clones for a given strain. For experiments using HaloTagged protein versions these are independent culturing events.

All NGS data (3C-seq, ChIP-seq) was deposited at GEO (GSE163573) together with respective processed data files (.txt). All other data is deposited at Mendeley Data (including replicates).

3C-seq samples are listed in Supplementary file 4. Reference to respective figure in manuscript, whether an independent biological replicate was prepared (_rep in “Additional info” column), whether a sample was sequenced deeper (_reseq in “Additional info” column) and number of valid reads are indicated. Raw and processed data files are available at GEO for both independent biological replicates, but in the manuscript map for one replicate is presented.

List of strains and respective genotypes are presented in Table 1 in the manuscript.

List of strains as they are presented in figures can be found in Supplementary file 2.

Figure 1

1. Scheme, N/A
2. Representative spotting assay of at least three independent biological replicates
3. Representative maps for at least two independent biological replicates
4. No replicates
5. Scheme, N/A
6. 3878 representative map for two independent biological replicates, 3882 single replicate

Figure 1 – figure supplement 1

1. Representative gel images for one of the replicates, plot: mean of three independent biological replicates.
2. No replicates
3. Representative gel image and quantification for one of the two replicates
4. No replicates
5. Representative spotting assays of at least three independent biological replicates. Two independently obtained clones were tested.

Figure 1 – figure supplement 2

1. Quantification of representative maps presented in Figure 1A
2. No replicates
3. No replicates
4. Representative spotting assay of at least three independent biological replicates. Two independently obtained clones were tested.
5. No replicates

Figure 1 – figure supplement 3

Representative plot for one of the replicates.

Figure 2

1. No replicates
2. Ratios of what is presented in A
3. Single replicate time course experiment
4. Single replicate time course experiment

Figure 2 – figure supplement 1

1. No replicates
2. No replicates

Figure 3

1. Representative maps for two independent biological replicates
2. Representative maps for two independent biological replicates
3. No replicates
4. Schemes, N/A

Figure 3 – figure supplement 1

1. No replicates
2. Schemes, N/A
3. No replicates
4. No replicates

Figure 4

1. No replicates
2. Representative map for two independent biological replicates
3. Representative maps for two independent biological replicates
4. Representative map for two independent biological replicates
5. No replicates
6. No replicates
7. No replicates

Figure 4 – figure supplement 1

1. Representative spotting assay of at least three independent biological replicates
2. No replicates
3. No replicates
4. No replicates
5. No replicates
