# Supplementary material for: A low Smc flux avoids collisions and facilitates chromosome organization in Bacillus subtilis
Source: eLife. 2021 Aug 4;10:e65467. doi: 10.7554/eLife.65467 (PMC8357415; doi:10.7554/eLife.65467)
Supplement: Supplementary file 2. [file elife-65467-supp2.docx]

**List of strains sorted according to relevant figure panels and ordered in the way they are presented.**

Figure 1

1. N/A
2. 1002, 3425, 3426, 3427, 3428, 2093, 1007, 1050, 3429, 3430, 3431, 3432, 3787
3. 1002, 3425, 3426, 3427, 3428, 2093

3790, 3801, 3802, 3803, 3804, 3932

1. 1018, 2352
2. N/A
3. 3878, 3882

Figure 1 – figure supplement 1

1. 1711, 4798, 2210
2. 1002, 3805, 3790, 3427, 3878, 3803, 1050, 4100, 1489
3. 4837, 4867, 4869, 4838
4. 2090, 2092
5. 1002, 1007, 1050, 2352, 3785, 2090

1002, 1007, 1050, 2092, 3786, 2093, 3787

Figure 1 – figure supplement 2

1. 3790, 3801, 3802, 3803, 3804, 3932
2. 3674, 3791
3. 3798
4. 1002, 1007, 1050, 3790, 3840, 3801, 3841

1002, 1007, 1050, 3802, 3842, 3803, 3843

1002, 1007, 1050, 3804, 3844

1. 3770, 3863, 2934, 3636, 3878

Figure 1 – figure supplement 3

1471, 1541, 1542,1543,1544

Figure 2

1. 1002, 3427

3790, 3803

1. 1002/3790, 3427/3803
2. 3026, 3216
3. 3026, 3216

Figure 2 – figure supplement 1

1. 1002, 3427

3790, 3803

1002/3790, 3427/3803

1. 3026, 3216

Figure 3

1. 3790, 3879, 4090
2. 3805, 4083, 4091
3. 3805/3790, 4083/3790
4. N/A

Figure 3 – figure supplement 1

1. 3805
2. N/A
3. 3815, 3790, 3805, 4083, 4091
4. 3815, 3790, 3805, 4083, 4091

Figure 4

1. 4100, 1002, 4143
2. 4100 (inset: 1002)
3. 4143, 4146
4. 4137 (inset: 3805)
5. 4100/4143, 4100
6. 4137/4143, 4137
7. 4152, 4427

Figure 4 – figure supplement 1

1. 1002, 4100, 1007, 3815, 3790, 4143, 3879, 4146, 3805, 4137
2. 4100, 4143, 4137
3. 4143/3790, 4143, 3790
4. 4100, 4143, 4137
5. 4152, 4427
