# Supplementary material for: A low Smc flux avoids collisions and facilitates chromosome organization in Bacillus subtilis
Source: eLife. 2021 Aug 4;10:e65467. doi: 10.7554/eLife.65467 (PMC8357415; doi:10.7554/eLife.65467)
Supplement: Supplementary file 3. — Key plasmid maps for generating the strains are deposited in Mendeley Data DOI:10.17632/kvjd6nj2bh.2. [file elife-65467-supp3.docx]

**Details of strain construction**

**General information**

Strains were prepared using allelic replacement with respective antibiotic selection on SMG (5 ug/ml kanamycin, 50 ug/ml spectinomycin, 8 ug/ml tetracycline, 5 ug/ml chloramphenicol or a combination of 0.4 ug/ml erythromycin and 10 ug/ml lincomycin).

Final plasmid sequence files are available in Mendeley Data.

**Engineering *parS* sites in *B. subtilis***

To adapt the number of *parS* sites in *B. subtilis* strains, congression was employed to avoid tagging the *parS* locus with an antibiotic resistance cassette. For congression, two DNA fragments targeting distinct loci are co-transformed and with certain probability co-integrated into the recipient genome. Antibiotic selection or prototrophic selection is based on the modification of the *smc-ftsY* locus or *trpC* locus, respectively using targeting dedicated vectors unrelated to *parS* loci.

To generate the *parS* targeting vectors, PCRs were performed on purified genomic DNA from either BSG336 [*PY79: Δ8-parS*, BNS1657 from (Sullivan et al., 2009)] or BSG1002 (*1A700, smc ftsY::ermB, trpC2*, wild-type strain from the Gruber Laboratory bacterial strain database).

Primers were designed in such a way that they allowed for amplification of a PCR product with 500 bp homology region on each side of the *parS* site. PCR products were cloned into an acceptor vector using Gibson Assembly and the insert was sequenced (Sanger sequencing).

To engineer *parS359* at *parS334* position, overlapping primers within the *parS334* were used to mutate the *parS334* sequence (5’-TGTTACACGTGAAACA-3’) into *parS359* sequence (5’-TGTTTCACGTGGAACA-3’). The amplified fragments were combined into a targeting vector by BsaI Golden Gate Shuffling using pSG436 as acceptor vector.

Next, standard *B. subtilis* transformation by starvation protocol was followed except that the cells were co-transformed with a mix of two targeting vectors: 10 ng of plasmid targeting either *trpC2* locus (pSG221 pUC19 'trpC') or *smc* locus allowing for marker switch (pSG001 pUC18 Smc locus with ErmB, pSG002 pUC18 Smc locus with SpecR) and 90 ng of plasmid targeting a selected *parS* locus.

Positive candidates that either became prototrophic (if transformed with pSG221, grew on SMG -trp) or were resistant to erythromycin/spectinomycin (if transformed with pSG001/pSG002, respectively), were later checked for presence of wild-type or mutated *parS* sites as indicated in the table:

|  | ***parS*** | ***mtparS* in BSG336** | **how checked?** |
| --- | --- | --- | --- |
| **1** | 4 | XhoI site present, 16bp del | PCR and XhoI digest |
| **2** | 15 | 14bp inserted, 16bp del | PCR and melting curve analysis |
| **3** | 40 | GCCG inserted, 14bp del | PCR and melting curve analysis |
| **4** | 334 | CGGCCG inserted, 16bp del | PCR and melting curve analysis |
| **5** | 354 | XhoI site present, 16bp del | PCR and XhoI digest |
| **6** | 355 | mtparS GGTCTCCCGGCTC | PCR and melting curve analysis |
| **7** | 356 | XhoI site present, 16bp del | PCR and XhoI digest |
| **8** | 359 | BsaI site present | PCR and BsaI digest |

As needed, successive rounds of congression were performed to eliminate or introduce multiple *parS* sites.

**Engineering the strains with inducible ParB**

A promoter of a constitutive, highly expressed gene *hbs* was coupled to a theophylline aptamer to regulate expression of *parB*. Region directly upstream of *hbs* was amplified from BSG1002 and cloned into a vector plasmid. The reverse primer (see sequence below, STM313) introduced the 3’ theophylline aptamer sequence at the end of the promoter region of *hbs*. 5’ sequence was similarly introduced using PCR (see sequence below, STM108) when amplifying the *parB(mtparS359)* coding sequence from pSG151 (pPL82PspacHY-Spo0J(mt*parS*)). Both plasmids were combined into a targeting vector by BsaI Golden Gate Shuffling using pSG1431 (pDG3661 *amyE::cat* BsaI-R1-RU acceptor) as acceptor vector.

STM313 (reverse in *Phbs* including 3’ part of theophylline aptamer sequence marked in bold)
5’-gttacatctagaggtctcacatc**aagacgatgctggtatcaccggtacct**agtaacacatataaaaagcc-3’

STM108 (forward in *parB* including 5’ part of theophylline aptamer sequence marked in bold)

5’- gttacagaattcggtctcagatg**cccttggcagcaccctgctaaggaggcatcaag**atggctaaaGGCCttggaaa-3’

**Engineering the strains for expression of increased amounts of Smc-ScpAB**

Strain with an additional copy of *smc* together with its endogenous promoter at *amyE* locus was used as a receipient (Bürmann et al., 2013). We introduced the extra copy of *scpAB* in between *ywcE* and *qoxD*, region similarly distant from the origin as *amyE*, on the other arm of the chromosome.

*scpAB* together with their endogenous promoter were amplified from BSG1002 and introduced into the vector plasmid. Two plasmids with homology region of *ywcE* and *qoxD* were also generated. All plasmids were combined with spectinomycin cassette into a targeting vector by BsaI Golden Gate Shuffling using pSG1525 (pET-Gate2 *mazEF*) as acceptor vector.
